# Designing a National Veterinary Prescribing Champion Programme for Welsh Veterinary Practices: The Arwain Vet Cymru Project

**DOI:** 10.3390/antibiotics10030253

**Published:** 2021-03-03

**Authors:** Gwen M. Rees, Alison Bard, Kristen K. Reyher

**Affiliations:** 1 Institute of Biological, Environmental and Rural Sciences (IBERS), Aberystwyth University, Penglais, Aberystwyth SY23 3DA, UK; 2 Bristol Veterinary School, University of Bristol, Langford BS40 5DU, UK; alison.bard@bristol.ac.uk (A.B.); kristen.reyher@bristol.ac.uk (K.K.R.)

**Keywords:** antimicrobial stewardship, veterinary, complex intervention

## Abstract

Antimicrobial use in agriculture has been identified as an area of focus for reducing overall antimicrobial use and improving stewardship. In this paper, we outline the design of a complex antimicrobial stewardship (AMS) intervention aimed at developing a national Veterinary Prescribing Champion programme for Welsh farm animal veterinary practices. We describe the process by which participants were encouraged to design and deliver bespoke individualised AMS activities at practice level by forging participant “champion” identities and communities of practice through participatory and educational online activities. We describe the key phases identified as important when designing this complex intervention, namely (i) involving key collaborators in government and industry to stimulate project engagement; (ii) grounding the design in the literature, the results of stakeholder engagement, expert panel input, and veterinary clinician feedback to promote contextual relevance and appropriateness; and (iii) taking a theoretical approach to implementing intervention design to foster critical psychological needs for participant motivation and scheme involvement. With recruitment of over 80% of all farm animal practices in Wales to the programme, we also describe demographic data of the participating Welsh Veterinary Prescribing Champions in order to inform recruitment and design of future AMS programmes.

## 1. Introduction

Antimicrobial resistance (AMR) is a global One Health challenge of great significance [1]. The World Health Organisation describes AMR as a global health and development threat requiring urgent multisectoral action [2]. While the development and transmission of AMR is complex and not yet fully understood, antimicrobial use is known to be a major driver of resistance and there is broad consensus that antimicrobial stewardship (AMS) is a key component in addressing the issue [2,3,4]. Indeed, “the critical role of antimicrobial stewardship in tackling the problem of AMR is reflected in its inclusion as a key action in the UK five-year antibiotic resistance strategy” [5]. Extensive AMS programmes are commonly seen in human healthcare settings [4,6,7] and, although they form a part of many national and global AMR action plans [2,3,5], their implementation in veterinary practice remains sporadic and small scale [8].

Antimicrobial use in agriculture has been identified as an area of focus for reducing overall antimicrobial use and improving stewardship [3]. In the UK context, recent efforts have led to a decrease in overall antimicrobial use in food-producing animals of 45% since 2015 [9]. These reductions have been broadly industry-led, with industry bodies recognising a consumer demand for responsible antimicrobial use and an increasing political focus on the issue [10,11,12]. Responsible prescribing is defined by the UK AMR 5-Year National Action Plan as “The use of antimicrobials in the optimal way, for the right pathogen, at the right dose, for the right duration, for the treatment or prevention of infectious disease.” [12].

In Wales, agriculture, animal health, and animal welfare are devolved policy areas over which the Welsh government has legislative powers [13]. AMR has been a policy focus in recent years, with the establishment of an Animals and the Environment AMR Delivery Group leading to the publication of the Welsh government’s five-year AMR Implementation Plan [11]. This plan includes the key focus areas of improving standards of antimicrobial selection and prescribing, as well as improving standards of antimicrobial supply. AMS has been recognised as a vital component of national AMR strategies, although there is work to be done to improve implementation [5]. The agricultural industry represents a proportionally greater percentage of the national economy in Wales than it does for the UK as a whole, and the majority of Welsh farming is based on beef, sheep, and dairy production [14]. As such, the health and welfare—and related antimicrobial prescribing—of cattle and sheep, could be argued as being of relatively greater significance in Wales than the rest of the UK.

AMS programmes are complex interventions consisting of several interacting and inter-relational components, which present challenges to those designing, implementing, and analysing such programmes [15]. Successful design requires evaluation of the available evidence, engagement with theory and a good theoretical understanding of how an intervention may cause change [16]. A recent systematic review found the use of theory, engagement of end users, identifying barriers, and selecting appropriate intervention components to be key elements of the successful design of interventions for changing healthcare professionals’ behaviour [17]. Additionally, involving stakeholders, understanding the intervention context and considering implementation in a “real world” setting have also been seen as essential principles for consideration [18].

The purpose of this paper is to outline the development and implementation of a national AMS scheme for farm animal veterinary practices through the establishment of a network of Veterinary Prescribing Champions (VPCs) as part of the wider Arwain Vet Cymru (AVC) programme in Wales. Arwain Vet Cymru is a collaborative initiative, which aims to train and support a national network of Veterinary Prescribing Champions across Wales to improve antibiotic prescribing in cattle and sheep. The project is participatory in approach, aiming to empower veterinary surgeons to develop and implement bespoke stewardship interventions, as well as share experiences and ideas. Both development and implementation of this scheme were informed by the self-determination theory (SDT), a broad theory of human motivation covering elements of interpersonal dynamics, goals and motives, individual differences, psychological needs, and psychological well-being [19]. SDT explicitly recognises that some behaviours are not intrinsically appealing and that the salient question when considering behaviour change is how to motivate individuals to value, self-regulate and (without external pressure) carry out and maintain, such behaviours. As such, SDT is particularly pertinent to the context of AMS, as it considers not just how and whether AMS behaviours are likely to be enacted, but the mechanisms by which these behaviours can become self-directed and, thus, maintained over time—elements critical to a national AMS scheme.

### Aim and Objectives

As the Veterinary Prescribing Champion Network is a novel intervention—with similar programmes now being considered for England and Scotland—this paper aims to inform future national stewardship programmes about its design, methodology, and enactment, providing a much-needed evidence base for future complex interventions in the veterinary sphere. Specific objectives are to examine:

- The process through which this national AMS scheme was appropriately contextualised, involving the integration of complementary knowledge pathways in the development of intervention goals;
- How intervention goals were subsequently grounded within a theoretical framework, by identifying operational SDT conditions and associated guiding principles relevant and applicable to VPC participation; and
- How the individual components of the AVC programme can lead to improved prescribing practice.

## 2. Methods

The study obtained ethical approval from the University of Bristol Health Sciences Research Ethics Committee, Reference 99522.

### 2.1. Study Setting

Antimicrobial use in Wales is regulated by the Veterinary Medicines Directorate, an executive agency of the Department for the Environment, Food, and Rural Affairs. All antimicrobials used in food-producing animals are prescription-only medicines, which can only be prescribed by veterinary surgeons to animals “under their care” [20]. Farmers in Wales are in the relatively privileged position of being able to store antimicrobials on farm for use at a later date [21]. There is a requirement to maintain purchase and use records, although there is evidence that these records may not always be accurate [22]. Veterinary practices in Wales that provide farm animal services are members of one of two Veterinary Delivery Partnerships, established to allow the delivery of government tuberculosis testing across Wales. Practices are otherwise separate and private business entities, with farmers able to choose their veterinary practice freely. There are approximately 50 separate veterinary practices providing farm animal services in Wales, although some of these are located along the border in England.

### 2.2. Theoretical Basis

The AVC intervention aimed to facilitate a professional environment that would inspire VPCs to engage with and endorse the network and their new AMS behaviours. Given the disparate nature of current antimicrobial prescribing and stewardship in veterinary practice, it was recognised that each participant’s context and behavioural opportunities would likely be different, and a one-size-fits-all approach to AMS was unlikely to be effective. This intervention was therefore founded on the selection, adoption, and implementation of AMS behaviour changes by AVC participants themselves, through participant involvement in the scheme cultivating a prescribing “champion” mindset to cement their intention to design and implement an AMS intervention within their own professional environments. In considering this target behaviour change through the lens of the widely used COM-B behaviour system (capability, opportunity and motivation) and the associated Behaviour Change Wheel [23], it was clear that achieving this goal necessitated a focus on delivering an intervention design that engaged core motivational drivers of individual AVC participants with regards to their engagement with AMS knowledge, principles, and activities. To this end, an evidence-based theoretical perspective was sought to inform the AVC process and activities with respect for—and targeted attention towards—fundamental VPC motivational needs. Few frameworks on motivation have spurred as much research as SDT, with a recent conceptual and empirical meta-analysis supporting key premises within the theory [24].

SDT identifies distinct types of motivation that are key to understanding how—and whether—behaviour becomes internalised by individuals and stimulates personal growth and change. The most fundamental distinction is between intrinsic motivation, which refers to carrying out a behaviour because it is inherently enjoyable or interesting, and extrinsic motivation, which refers to carrying out a behaviour because it leads to a separable outcome or instrumental value [25]. For example, a veterinary surgeon who spends her spare time reading a paper on responsible prescribing practices, purely because she is curious about the topic, does so because she is intrinsically motivated, whilst her colleague who carries out the same behaviour only because it has been mandated by their boss is extrinsically motivated.

Extrinsic motivation can be further classified by its underpinning reasons or goals, forming a continuum from internalised and agentic extrinsically motivated states to those that are more motivationally impoverished and externalised [25]. Those extrinsic behaviours that are more internalised (i.e., in line with an individual’s closely held beliefs or values) are likely to be associated with better quality of engagement, more positive self-perception and greater persistence than those behaviours that are more externalised (i.e., those carried out due to external punishments and rewards or a focus on approval from others via, for example, pride, shame or guilt) [24,25]. As such, for the veterinary surgeon reading about responsible prescribing because his boss requires it, if he also views the activity as valuable in developing his professional knowledge and identity, he will be more effectively engaged than if he acts purely to avoid guilt or a reprimand.

Where the premise of this intervention was to encourage individuals to carry out behaviours that might not have been intrinsically motivated (otherwise, no intervention would arguably have been necessary), we believed promoting conditions that allowed VPCs to feel more in control—and to express internalised motivation in their AVC engagement and chosen AMS behaviours—to be critical to intervention success, given associated benefits in learning, engagement, creativity, and personal commitment [25]. SDT identifies three universal psychological conditions that—across cultures—are critical to promoting internalised forms of extrinsic motivation in individual behaviour: the needs to feel competence (perceived self-efficacy), autonomy (a sense of choice, being the origin of one’s own behaviour), and relatedness (feeling understood and cared for by others) [26,27]. Significant consideration was therefore paid to fostering these conditions in all aspects of programme delivery to promote VPC self-direction in AVC activities and resulting AMS behaviour change goals (Table 1).

### 2.3. Engagement through Key Collaborators

The AVC project—which also includes quantitative antimicrobial use data collection and animal health planning schemes alongside the intervention—represents a collaboration between Bristol Vet School, Welsh government’s Office of the Chief Veterinary Officer, the industry-controlled farmer cooperative Welsh Lamb and Beef Producers and the South Wales and North Wales Veterinary Delivery Partners, Iechyd da and Milfeddygon Gogledd Cymru, respectively. Development of a network of Veterinary Prescribing Champions is one part of the wider AVC project, which is aimed at addressing antimicrobial resistance in Welsh agriculture. This includes work to develop technology for improving the accuracy of medicine use recording by farmers, led by Welsh Lamb and Beef Producers, and benchmarking veterinary practice antimicrobial use, led by Iechyd Da. By engaging these key collaborators, the AVC intervention was supported by leading academic, governmental and industry representatives able to engage with potential participants and encourage active involvement in the programme. Each collaborator contributed to engagement in the following ways: The Office of the Chief Veterinary Officer was able to use established communication channels to encourage participation in an official capacity, with the Chief Veterinary Officer for Wales endorsing the programme and encouraging veterinary surgeons to take part. Welsh Lamb and Beef Producers are responsible for farm quality assurance schemes in Wales and, therefore, are a familiar industry body that Welsh farm animal veterinary surgeons understand to represent farmers’ interests, which helped improve engagement. The Veterinary Delivery Partners were able to contribute to active recruitment by disseminating details of the project to their veterinary practice members through formal networks. The project lead (GR) had also worked as a farm animal veterinary surgeon in Wales and, therefore, was able to combine these formal recruitment pathways with informal networks to further promote engagement.

Participant demographic data were collected through an online questionnaire at the time of initial recruitment and registration.

### 2.4. Designing a National Stewardship Programme

**Figure 2:**
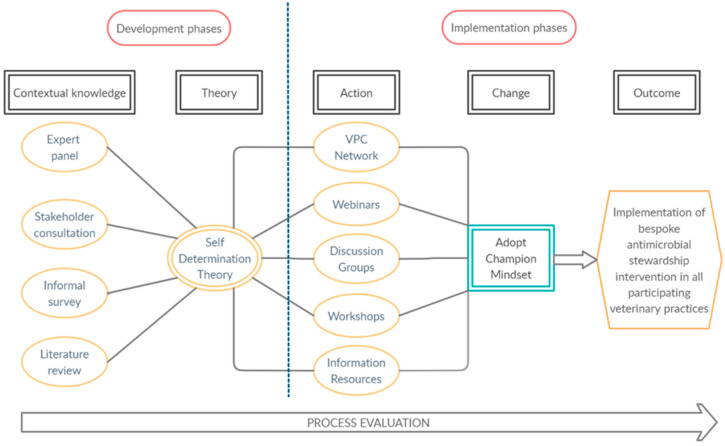
Design map of the Arwain Vet Cymru program, identifying the key phases of the design and implementation process.

The development of the AVC intervention model occurred in two phases (Figure 2). Firstly, identifying critical elements of the intervention—through the integration of the four knowledge pathways representing subject experts, relevant stakeholders, practicing veterinary surgeons, and the current evidence base for effective interventions targeting prescribing practice—enabled the design of a context-specific and appropriate intervention. Secondly, grounding the delivery of this intervention within the SDT theoretical framework—by identifying operational SDT conditions relevant and applicable to AVC participation—allowed for an understanding how the intervention was proposed to engage VPCs’ internalised motivation.

#### 2.4.1. Phase One: Contextual Knowledge

Four knowledge pathways were explored to appropriately contextualise the aims of the AMS program for VPCs in Wales.

Relevant stakeholders: key stakeholders were identified in the areas of veterinary professional regulation, specialist veterinary membership organisations, farming body representatives, government policy departments, and human public health. Stakeholders included the British Veterinary Association’s Welsh Branch Council, Welsh government’s AMR in Animals and the Environment Delivery Group, the Sheep Veterinary Society, the British Cattle Veterinary Association, Public Health Wales, and the National Farmers Union, among others. These stakeholders were contacted and invited to input into the design of the new national stewardship programme. Stakeholders involved in ongoing animal health projects in Wales were contacted in order to coordinate efforts and avoid duplication.

Practicing veterinary surgeons: practising farm animal veterinary surgeons in Wales were informally surveyed by the Veterinary Delivery Partners in order to identify key issues they felt important to be included in the design of the programme. This took the form of utilising existing communication networks between farm animal veterinary surgeons, including email and WhatsApp communications, to invite suggestions for stewardship intervention strategies and feedback on current policy.

Expert input: a broad range of expertise was available through the University of Bristol’s “AMR Force” multidisciplinary research group, consisting of clinical veterinary practitioners, epidemiologists, veterinary academics, and social scientists. By drawing upon this expertise, intervention design was informed by the current research landscape and areas of clinical importance in order to focus on identified areas of key importance to research and clinical practice.

Literature review: an extensive literature review examining (i) complex intervention design theory; (ii) antimicrobial prescribing in agriculture; and (iii) AMS interventions was conducted. This review provided an evidence base for the intervention design, identified potential barriers, and enablers to stewardship in the veterinary context and highlighted known areas of high antimicrobial use for specific focus.

#### 2.4.2. Phase Two: Integrating Theory

The second phase of AVC intervention design aimed to foster the motivational internalisation of AVC activities and AMS change for participating VPCs. To ensure VPCs’ motivation was cultivated in this internalised, agentic form, active integration of the psychological needs highlighted within SDT was critical. Namely, the need for VPCs to feel AVC activities and selected AMS change(s) (i) enhanced their competence (perceived self-efficacy); (ii) supported their autonomy (a sense of choice, being the origin of one’s own behaviour); and (iii) promoted their sense of relatedness (feeling understood and cared for by others) [26]. Operational conditions for these psychological needs have been detailed for consideration in the design of SDT-informed interventions of this kind [27]. These conditions were adapted to create guiding principles for the AVC intervention design (Table 1) informing the selection, content, and thoughtful delivery of activities within the AVC training schedule, as highlighted in Results.

## 3. Results

### 3.1. Participation of VPCs

A total of 43 farm animal veterinary surgeons were recruited to the AVC project from March 2020, representing 41 veterinary practices across Wales. Participants were offered no incentives for taking part in this study, although the training could be counted towards mandatory continuing professional development requirements of UK practicing veterinary surgeons. Out of the 50 Welsh practices involved in farm work (defined as practices with Official Veterinarians registered with the Veterinary Delivery Partners), nine did not take part in the programme. Of these, five stated that they did not do sufficient farm work within Wales to make participation worthwhile, one practice withdrew from the programme due to increased workload relating to the Coronavirus Disease (COVID-19) pandemic and no response was received from three practices. Demographics of participants can be seen in Table 2.

Fifty-eight percent of the veterinary surgeons participating were either business partners or directors, consultants, or clinical directors, with the remaining 42% identifying as salaried assistants. Half of the participants had been graduated for >20 years, with only 5% having graduated fewer than five years prior to the programme beginning. Participants had a diverse range of interests across the spectrum of farm animal clinical work, with similar proportions interested in dairy, sheep, beef, mixed practice, and smallholder work. Twenty-six practices belonged to the South Wales Network, and 17 practices belonged to the North Wales Network. Eight practices were based over the Wales–England border, but served a significant number of Welsh farms. Nineteen participants (44%) were female and the remaining participants male.

The key barriers to implementation found so far in the AVC project can be best characterized as time constraints for participants and concern that restricting antimicrobial prescribing may lead to farming clients sourcing medicines elsewhere. However, despite a focus on these barriers during group discussions, they have not impacted significantly on participation in the programme to this point.

### 3.2. Defining the AVC Intervention Structure

Four knowledge pathways, as outlined in Methods, determined the structure, and focus of the overall intervention:

Stakeholder engagement: stakeholder response was positive, with all those contacted recognising the need for an AMS programme in Wales. Topics that emerged as important from the stakeholder engagement included a focus on responsible antimicrobial sales practices, the need for greater communication and collaboration between veterinary practices within a region, supporting veterinary surgeons to make responsible prescribing decisions and improving knowledge of relevant legislation and guidance.

Practicing veterinary surgeons: an informal survey of the needs and desires of farm animal veterinary surgeons in Wales indicated that they had similar areas of concern and focus as those identified by stakeholders. Of particular importance was the issue of responsible antimicrobial sales practices and improving communication between practices. Practising veterinary surgeons also outlined an interest in behaviour change principles, and how they could be applied when encouraging farmers to use medicines responsibly.

Expert input: interdisciplinary research group meetings outlined several key areas that were viewed as important in the design of this intervention. These included improving knowledge of the legal aspects, professional regulations, and industry guidelines surrounding prescribing, the principles of evidence-based veterinary medicine and the importance of participatory approaches to change.

Literature review: grounding the design in the theory of behaviour change and complex interventions in healthcare was identified as very important to the programme’s success. Reviewing the literature indicated that the use of so-called “Champions” in health care interventions had been successful in other settings. The literature also highlighted the benefit of building sustainable communities of practice for complex healthcare interventions, and of combining education and training resources with reflective exercises and goal setting.

By combining the results of these four knowledge pathways, AVC’s design was focussed around addressing the following key areas:

- Recruit and train one VPC from each farm animal veterinary practice in Wales.
- Improve VPCs’ knowledge of AMS, the evidence base for prescribing decisions and the evidence base for legal and regulatory frameworks, human behaviour change, and species-specific considerations.
- Foster a sense of group identity as well as of community and collaboration between Champions.
- Encourage Champions to disseminate AMS messages within their practices.
- Facilitate the autonomous development, by each individual participant, of individual practical, fit-for-purpose stewardship interventions at each participating practice.

### 3.3. Enactment of the AVC Network: Combining Intervention Goals and Theoretical Drivers

The overall design of the implementation can be seen in Figure 2, and the training schedule can be seen in Table 3. Initially, implementation of the programme was designed to consist of several in-person meetings of all VPCs over the course of the first year. However, following the COVID-19 global pandemic and subsequent lockdown in the UK in March 2020, combined with the uncertain future of large gatherings, it was necessary to reimagine the AVC process in an entirely online format in early 2020. Each element of this online format within the AVC process will be discussed with reference to the operational conditions of SDT (Table 1) identified as critical guiding principles of the AVC intervention design.

### 3.4. Webinars

In order to address the goal of improving VPCs’ knowledge of the key areas of AMS identified in the knowledge pathways outlined above, an educational programme of six webinars was included in the overall design. Expert speakers were invited from a range of academic institutions, with content informed by the literature review and expert panel meetings along with veterinary and stakeholder engagement. Six one-hour webinars were co-designed with the speakers. These webinars were broadcast weekly on Wednesday afternoons, the day identified during recruitment as the best time for VPCs as routine tuberculosis testing does not usually occur on this day. Participants were given the opportunity to attend webinars during the live broadcast or to watch recordings asynchronously, at their convenience.

A brief description of the content of each webinar is outlined in Table 3. Briefly, webinars covered topics such as Welsh AMR policy, the concept of AMS, legislation and guidelines relevant to prescribing, behaviour change theory, evidence-based veterinary medicine, antimicrobial use benchmarking across the different species and a selection of case examples from practices that had successfully implemented various AMS schemes. These topics were selected based on the key areas identified in the literature review, stakeholder engagement, expert input, and informal survey of practicing veterinary surgeons, as outlined in Section 3.2.

The format and delivery of these webinars was chosen to actively promote operational conditions within SDT to enhance VPC engagement (Table 1). The provision of instrumental and practically relevant AMS training—in addition to clarifying VPCs’ expectations of their involvement in the AVC Network—promoted support for VPC-perceived competence. Additionally, focusing on promoting VPC self-endorsement of AVC activities through provision of a variety of rationales from well-respected, expert speakers whilst providing choice in how webinars were accessed by participants (i.e., synchronous or asynchronous) embedded key attributes of autonomy support.

### 3.5. Discussion Groups

To develop a sense of community, collaboration, and group identity, informal online discussion sessions were held every third week of the nine-week training timeline (Table 3). VPCs were divided by region into North Wales and South Wales groups. Participants were given a choice of which group they wished to belong to, since those working in mid-Wales may have identified more strongly with a different region than might have been suggested geographically. Discussion sessions were hosted using online videoconferencing software and were facilitated by two researchers experienced in group facilitation (G.R. and A.B.).

Topics of discussion in each session were iterative and informed in part by the content of the previous webinars, in addition to topics raised during informal feedback and webinar question-and-answer sessions. These topics were guided by the facilitators, but were semi-structured in nature, allowing some freedom for participants to discuss issues they felt to be important at the time. Utilising interactive polling and small group breakout rooms, participants were asked to focus on and discuss specific areas related to veterinary prescribing before joining plenary discussion sessions where participants were able to share their views and discuss further with the whole group. Discussion group size varied from a minimum of five participants to a maximum of 17 participants between group meetings, and the facilitation of these groups was flexible in order to account for varying group size. Where discussion group size was greater than five, virtual “break-out rooms” were used, and participants were asked to discuss in small groups before returning to the main plenary discussion to report back on their discussions. This flexibility was important because the availability of participants to join discussion groups would vary depending on clinical veterinary duties on the day.

Discussion sessions enabled participants to outline the main challenges they perceived when considering implementing AMS programmes, along with exploring opportunities for change(s), and developing a sense of shared ownership over the outcomes of the project. Discussion sessions were also an opportunity to prepare Champions for the subsequent workshops. Promoting congruence with the tenets of SDT underpinned discussion session design. To cultivate a sense of autonomy for VPCs, attendance was made non-compulsory and VPCs chose their regional group allocation. The sessions were also opportunities for AVC facilitators to actively evoke and acknowledge VPCs’ feelings and agendas with regards to the breadth of potential interventions covered in the webinar sessions. This, in turn, further promoted autonomy through respect for the VPCs’ unique choices and intentions with regards to the AMS foci. To support VPCs’ competence, the discussion groups offered facilitators the chance to provide relevant and non-judgemental feedback on VPC perspectives on AMS foci, whilst allowing facilitators to shape participatory activities to also encourage positive peer-to-peer feedback. Finally, enhanced relatedness was achieved through a focus on evoking, exploring and understanding VPC perspectives, both by the facilitators and through targeted peer-to-peer activities, creating opportunities for promoting group empathy and attunement (a felt sense of union) between AVC participants.

### 3.6. Workshops

Two three-hour facilitated workshops were included in the design of the programme and followed on from the webinars and discussion groups in order to enable goal setting and the creation of action plans by VPCs, as outlined below. The first workshop was intended to allow VPCs to develop the knowledge and ideas gained during the webinars and discussion groups and distil these into actionable goals designed specifically for their practice context. VPCs were responsible for designing their own context-specific AMS intervention, relevant to their veterinary practice’s prescribing context. A second workshop, designed to inform policy, was included in order to allow VPCs the opportunity to contribute to the wider professional context with regards to matters of AMS.

#### 3.6.1. Stewardship Intervention Design Workshop

The stewardship intervention design workshop aimed to enable each participating VPC to design and develop their own personal action plan, as well as a stewardship intervention for their practice. Examples of the kind of action plans discussed include:

- Reorganise the practice veterinary medicine dispensary to make certain antimicrobials more difficult to reach and/or more easily identified as second or third choice.
- Schedule training and improve communication with veterinary reception and dispensing staff at the practice to ensure all staff members are delivering a unified message around antimicrobial prescribing and dispensing.
- Begin to benchmark antimicrobial use among practice farms and include discussion of antimicrobial use in annual herd or flock health planning.
- Introduce on-farm medicine cupboard “health checks” into the annual herd or flock health planning.

This workshop was run by an experienced participatory action research facilitator and co-facilitated by two experienced facilitators familiar to the VPCs (G.R. and A.B.). Participants were asked to set goals and create action plans outlining how they would implement their stewardship intervention according to the SMART framework (Specific, Measurable, Attainable, Relevant, Timely) [30]. Structured discussions utilising online fora and breakout rooms allowed VPCs to consider their plans with their peers, helping to identify potential barriers to implementation and possible solutions by drawing on their collective experiences.

Central to the concept and design of this workshop was fostering VPCs’ sense of autonomy in their AMS roles. Workshop activities consolidated VPCs’ own ideas for an AMS intervention strategy depending on what they envisaged for their own practice context, whilst a primary facilitator experienced in non-directive, participant-led workshops emphasised the ethos of VPC choice and self-endorsement throughout. Workshop activities also aimed to encourage VPCs toward the choice of an optimal AMS challenge (i.e., not too easy nor too difficult) for their circumstances and skill set, to drive competence-infused practice change. Relatedness was embedded within workshop activities through, for example, informal and personal introductions in each workshop to foster rapport, by offering attending VPCs opportunities to vocalise fears, concerns, and thoughts on intervention interests for peer validation, and facilitating peer-to-peer exploration, reflection, and group feedback on personal perspectives of AMS activities and policy within Wales. Together, activities of this kind sought to foster empathy and union (attunement).

#### 3.6.2. Policy Workshop

This workshop was designed to allow VPCs the opportunity to inform AMR policy at the national level. Participants were encouraged to identify important areas of focus, outline the policy support required to enable them to be responsible prescribers, and construct practical solutions to help address some of the barriers identified in the AVC project. This workshop was created with the support of Welsh government, who agreed that outcomes would be presented to the Welsh government’s Animals and the Environment AMR Delivery Group.

This policy workshop offered VPCs the chance to develop a sense of personal influence over policy decisions impacting their profession; thus, engendering a feeling of self-endorsement critical to autonomous engagement. The premise of the workshop—highlighting the unique role of the AVC network as a valued voice in determining AMR strategy in Wales—emphasised the importance of this group influence of AVC Champions thus forging the relatedness of group members further. Providing another practical opportunity for VPCs to explore and construct solutions to AMS challenges, elevated to the national perspective within Wales, was a final training opportunity for VPC competence development.

### 3.7. Stewardship Intervention Implementation

The initial AVC programme outlined above required a time commitment of around 15 h by the VPCs. Following on from the workshops, VPCs are expected to disseminate the AMS messages to and implement their co-designed, individual AMS plans in their respective practices. This will lead to 41 different AMS schemes being implemented—one at each participating practice—beginning in January 2021. Participants will be asked to complete monthly reports outlining the implementation of their stewardship scheme as well as provide feedback on ease of implementation, relevant actors involved, scope of the changes, outcomes observed, and barriers encountered. Overall practice prescribing behaviours will be evaluated through a longitudinal prescribing audit.

Throughout implementation, AVC facilitators will manage VPCs through a continued ethos of avoiding directive, coercive, or authoritarian approaches, first and foremost emphasising VPC autonomy in the enactment of practice-based interventions and how VPCs choose to engage with the AVC Network, and support staff throughout this process. Practices will be supported by the project as required, with AVC facilitators prioritising trust in VPC competencies in overseeing the implementation of these intervention choices, with a focus on responsive management determined by VPCs themselves. It is hoped that the attunement developed from peer-to-peer activities within the AVC Network (through discussion groups and workshops) will also be an avenue of relatedness support for participating veterinary surgeons during this implementation process.

## 4. Discussion

In a complex healthcare intervention such as this, multiple interconnected elements all inform and affect each other. The AVC design process included stakeholder engagement, reviewing current literature, drawing on theory and understanding context as laid out by O’Cathain et al. [18]. As such, it is difficult to appraise each individual element of such a programme, and the entire intervention must be considered as a whole. However, by examining some of the principal domains of the intervention design (Figure 2), it is possible to explore how they informed—and became intrinsic to—the programme structure. By attempting to understand the context, theoretical basis, and implementation of the intervention, we can examine how and why VPCs within the AVC network might promote change through their AMS interventions and lead to more responsible use of antimicrobials.

Considerable effort was spent throughout the intervention process in engaging and communicating with farm animal veterinary surgeons in Wales. By grounding the design in the available literature and accessing informal feedback, key barriers to implementation could be identified and attempts to overcome them could be incorporated into the design from the outset. A recent scoping review found that knowledge, responsibility (the influence of peer behaviour) and the veterinary surgeon–client relationship represented significant barriers to AMS for cattle veterinary surgeons [31]. The AVC implementation design sought to address these barriers through education and building communities of practice. In Golding et al.’s exploration of veterinary surgeons’ beliefs about AMS, one perceived barrier to implementation was the concern that farming clients might simply change to a rival practice if denied the antimicrobial of their choice [32]. This was also identified by stakeholders and participants as a barrier to change. In response to this, the design of the AVC intervention included an emphasis on building a sense of common purpose between practices, encouraging open communication, and creating a community by incorporating informal discussion groups.

The demographic characteristics of the participants was hypothesised to play an important role in the likely success of the program. Experienced veterinary surgeons with a senior role in their practices were thought to have a greater degree of autonomy and authority with which to implement AMS interventions. The relatively few female participants (44%) compared with the 57% of females who make up the UK’s veterinary workforce was statistically significant (*p* = 0.0472) using the N-1 Chi-Squared Test for two proportions and may be explained in part by the increasing “feminisation” of the profession and the under-representation of female veterinary surgeons in senior roles [33]; recruitment of older, senior veterinary surgeons meant they were more likely to be male. It would be interesting to understand whether the gender ratio of such a participant group—and its representativeness of the wider study population—influences the effectiveness of the intervention. Further research into the role of gender in complex intervention implementation through realist evaluation principles is required [34].

In identifying a theoretical driver for AVC, full consideration of the intervention context was essential. Following the recommendations within COM-B [23], AVC design considered (i) the target behaviour; (ii) intervention options; and (iii) content and implementation options. The target behaviour within AVC was complex, with the aim of participants cultivating a prescribing “Champion” mindset and cementing their intention to design and implement an AMS intervention within their own professional environments. Fundamentally, the AVC goal was therefore to create the facilitative conditions for this mindset and practice change to occur. Intervention options from the Behaviour Change Wheel targeting training, environmental restructuring, and enablement appeared most appropriate for this purpose, influencing the multifaceted intervention design and the inclusion of webinars, discussion groups, and workshops informed by the four knowledge pathways [23]. Through consideration of how best to integrate these intervention foci effectively as drivers of capability, opportunity, and motivation with regards to a “Champion” mindset, a theoretical underpinning was sought to foster VPC engagement throughout.

Key to the aim of this intervention was the need for the “Champion” mindset to be sufficiently salient, psychologically, to drive VPC self-directed behaviour as VPCs are expected to implement their own AMS intervention in January 2021, following active engagement in the AVC scheme. Understanding the motivational factors that facilitate or undermine a sense of initiative and volition, in addition to the quality of performance, is central to SDT [19,23]. This theory therefore appeared uniquely adapted to the demands of the AVC scheme. The psychological conditions posited to encourage individuals to value, self-regulate and (without external pressure) carry out and maintain behaviour—competence, autonomy, and relatedness [26]—were adopted as guiding principles in the practical realisation of the intervention design. The strength of this intervention lies in having conducted a thorough assessment of: the behaviour change target in question, what might be needed to achieve this change, and where a theoretical underpinning resonating with the project aims might enrich implementation [35].

In the pursuit of forging individual identities within health interventions, the concept of using “Champions” as a means of motivating change in healthcare settings is not new. In Australia, Antibiotic Champions have been used to support an AMS campaign within Children’s Health teams [36]. Medical, veterinary and dental students in the UK can register to become Antibiotic Guardian Champions [37], and the UK’s National Health Service (NHS) has several Champion schemes, addressing such issues as social prescribing [38], diabetes [39], physical activity [40], perinatal metal health [41], and digital health [42]. In this programme, giving the participants an identity, as a VPC was a crucial part in developing a sense of community and leadership. Champions were representing the programme within their practices but were also representing their practice within the network.

Complementing this individual shift in perspective was the hypothesized creation of communities of practice, forging a group identity for AVC participants. The Situated Learning Theory [43], whereby professional learning occurs through interaction with peers and participation in practice, forms the basis for the concept of communities of practice. These are groups of people who interact on an ongoing basis in order to share expertise and deepen knowledge on an area of concern [44]. They have been utilised in healthcare settings as a means of improving performance and sharing knowledge “in response to the challenges of complex systems” [45,46]. By encouraging the development of a superordinate identity—in this case that of a national Prescribing Champion—alongside their professional identities as veterinary surgeons working in discrete private practice, it was intended that VPCs could overcome the professional barriers to AMS identified in the literature, as suggested by Bartunek et al. [47].

The inclusion of goal setting and action planning in this programme, through the intervention design workshop, allowed VPCs the opportunity to translate the knowledge and ideas gained during the initial training into defined, outcome-driven actions. By using the SMART framework [30], creating individual action plans based on overarching goals was expected to help narrow the intention–behaviour gap [48]. Literature establishes that planning within a particular context of who, when, where, and how is important when considering behaviour change [4]; indeed, it is at the heart of the theoretical underpinnings of the COM-B model [23]. Encouraging VPCs to consider these elements in their individual intervention designs will ideally facilitate AMS plans that appropriately echo tenets of the COM-B model, even in the absence of direct training on the intricacies of this model. A recent paper by Atkins et al. specifically called on National AMS intervention design to include goal setting and action planning, as they were areas identified as being under-represented in current AMS programmes [49].

Educational interventions have been shown to improve knowledge of pharmacovigilance [50] and prescribing competency [51] as well as to strengthen AMS [52,53,54], although the effects may be short-lived. Online learning as part of AMS programmes has been playing an increasing role [55] and online training of GPs has been shown to reduce antimicrobial prescribing for respiratory disease [56]. An online process also enabled the inclusion of a diverse range of external expert speakers who may not have been able to attend in more traditional in person provision, potentially improving the overall content. Another unintended but positive effect of online provision was the distribution of training sessions over the course of several weeks, interspersed with other activities, thus potentially consolidating the VPCs’ participation in the programme.

The Medical Research Council’s new guidance for developing and analysing complex interventions [57] highlights the importance of practical effectiveness—that is, whether the intervention works “in the real world”—as a key measure when evaluating complex interventions. In order to answer this question, process evaluation can use ethnographic and qualitative methodology in order to explore the impact of the intervention, identify any unintended consequences and be able to describe the experience of the participants who take part in any intervention programme. Through an ongoing process evaluation combining ethnographic exploration of implementation and quantitative measures of prescribing, the implementation of the AVC programme will be under continuous appraisal until completion (September 2021). Results of this evaluation will be published separately.

It remains to be seen whether the Arwain Vet Cymru project produces workable AMS interventions in clinical veterinary prescribing practice as predicted. While it is hoped that this complex intervention is successful in improving responsible prescribing practices, further empirical evidence is currently being collected in order to enable full conclusions to be drawn. Any unforeseen negative consequences are of course also important, and all outcomes are meaningful when informing future development of similar programmes, both in Wales and further afield.

### Limitations

While in an ideal world the design–evaluation–implementation process would occur in a relatively linear fashion and follow best-practice study design, practically this is not always possible. In this instance, the intervention took place in the context of political and industry-led pressure on the veterinary profession to improve prescribing, with an impact-led rather than research-led funding focus. As such, design, evaluation and implementation occurred in a more cyclical and iterative process in this study.

The establishment of this national Network of Prescribing Champions has been relatively labour-intensive, requiring high levels of ongoing engagement with key actors across many stakeholder groups. Participation in the project has involved around 15 h of time investment from participating VPCs, and the ongoing time commitment required to implement their action plans will be dependent on the complexity of the intervention each VPC has designed. The other stakeholders involved in the development of the AVC stewardship programme were not compensated for their time, and we believe their involvement to be motivated by a desire across the veterinary profession to improve antimicrobial prescribing both for the “greater good” and to improve the image of UK agriculture. Given the economic and political sensitivities of bringing individuals from separate, competing interests together to tackle a common concern, the very high level of recruitment to the programme is both surprising and encouraging. Establishing this pan-Wales network of highly motivated clinicians may make it possible to overcome some of the perceived barriers to change. The ongoing sustainability of the network—and its legacy after the end of the funded project—is an important area for development in the next stage of the programme. By moving to a self-sufficient model of participant-led network maintenance, it may be possible to continue the network beyond the lifespan of the project.

## 5. Conclusions

Designing a novel national AMS programme for farm animal veterinary surgeons requires several supporting factors. The applicability of this programme design to other parts of the UK and the rest of the world is difficult to predict; however, we believe that by focusing on a robust theoretical grounding and giving full consideration to the context of the intervention as evidenced in this paper, stewardship interventions can be improved worldwide. A favourable policy background, collaboration with key actors within the profession, stakeholder consultation, an emphasis on autonomy, and commitment to developing a sense of community have all helped to promote high levels of engagement in this voluntary national network of VPCs. Empirical data from both qualitative and quantitative process evaluations will help reveal the impact this type of complex intervention may have on AMS in rural veterinary medicine.

## Figures and Tables

**Figure 1:**
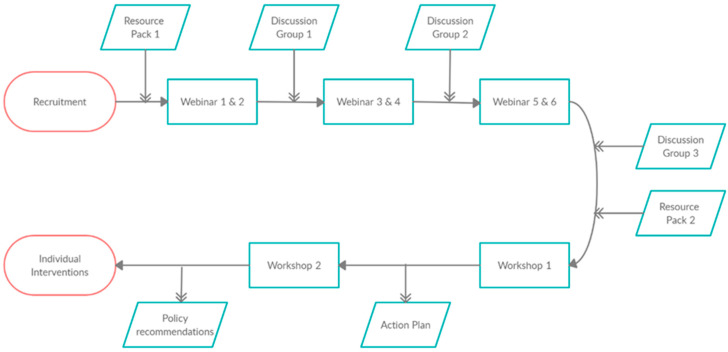
Flowchart showing the chronology of the inputs and outputs in the Arwain Vet Cymru programme.

**Table 1:** Operational conditions of self-determination theory posited by Silva, Marques, and Teixera [27] for consideration in intervention design and their adaptation to guiding principles for appropriate enactment within Arwain Vet Cymru.

SDT Construct	Operational Condition	Guiding Principle
Support for autonomy	Relevance	Provide a clear and meaningful rationale for both AVC and AMS activities throughout all inputs and training elements of the AVC programme (Figure 1), aiming to facilitate self-endorsement of activities by VPCs.
	Respect	Seek to actively acknowledge VPCs’ perspectives, feelings, and agendas within network activities. Thoughtfully integrate opportunities within the programme for individuals to contribute to, shape, and offer reflection on the intervention process, foci, and goals as they unfold.
	Choice	Embed engagement with AVC activities with a sense of choice wherever possible, by providing varied options for process engagement (i.e., in educational training and network meeting participation) and encourage VPCs to follow their own interests, ideas, and goals in the selection, adoption, and implementation of AMS intervention activities.
	Avoidance of control	Commitment by those leading AVC to avoid directive, coercive, or authoritarian management of VPCs within the network; ensuring this ethos leads to the selection of collaborative partners who contribute to practical programme delivery (such as external facilitators).
Support for competence	Clarity of expectations	Ensuring that through recruitment, inputs and training activities within the AVC programme (Figure 1) discussion of what to expect and what not to expect from AVC participation is facilitated. Set up processes that encourage the setting of realistic and achievable behaviour change goals by VPCs in their adoption and integration of AMS options.
	Optimal challenge	Seek to encourage VPCs to select behaviour change goals where the challenge of the activity is highly balanced with their ability to successfully perform the behaviour (i.e., the change is a good fit for their practice and context, is something that they have the appropriate skill set to enact, and that is neither too easy nor too difficult for the VPC to implement).
	Feedback	Ensure VPCs have the opportunity to access relevant and non-judgmental feedback on their practice interventions throughout design and implementation processes, both individually (through accessibility of contact with G.R. as project lead) and in-group meetings where this is facilitated peer-to-peer within the network (i.e., workshops and discussion groups).
	Skills training	Commitment to providing education, training, guidance and support in key areas of AMS as identified through knowledge pathways in Phase One of intervention design, to ensure VPCs feel adequately equipped to identify and set their own AMS behaviour change goal(s).
Support for relatedness	Empathy	Ensuring group meetings (discussion groups, workshops) offer opportunities for VPCs to explore and reflect on their colleagues’ perspectives at both peer-to-peer and group levels. Facilitate alternate perspective taking on any contentious issues if they arise within the group.
	Affection	Those coordinating the AVC scheme taking care to convey a sense of care and concern for participants prescribing and AMS challenges, in addition to genuine appreciation for VPC engagement.
	Attunement	Careful attention to, gathering knowledge about and responding to VPC perspectives both (i) by those coordinating the AVC scheme and (ii) facilitated peer-to-peer within the AVC network, to ensure VPCs needs to feel validated, accepted, affirmed, and significant within AVC are met [28], and to generate a felt sense of union with other VPCs in this process [29].
	Dedication of resources	Emphasising where and how AVC coordinators and wider project collaborators (industry, government) are investing time and energy into the scheme, in addition to creating project opportunities (workshops, discussion groups) where VPCs are connected by volunteering their time and energy to drive the momentum of AVC.
	Dependability	Ensuring VPCs feel that support is available to them via AVC in case of need on their AMS behaviour change journey, through guidance on how they can seek the input and advice of the project lead (GR) throughout.

Where SDT = self-determination theory, AMS = antimicrobial stewardship, AVC = Arwain Vet Cymru, VPC = veterinary prescribing champion, GR = Gwen Rees.

**Table 2:** Participant demographics of Veterinary Prescribing Champions (VPCs) enrolled on the Arwain Vet Cymru project in Wales.

Participant Characteristic	All VPCs		North Wales VPCs		South Wales VPCs	
All VPCs	43	100%	17	100%	26	100%
**Gender**						
Male Female	24 19	56% 44%	11 6	65% 35%	13 13	50% 50%
**Years qualified**						
<5 years 5–10 years 10–20 years >20 years	2 8 11 22	5% 19% 25% 51%	0 4 5 8	0% 24% 29% 47%	2 4 6 14	8% 15% 23% 54%
**Position in practice**						
Business Partner/Director Clinical Director Consultant Salaried Assistant	19 5 1 18	44% 12% 2% 42%	7 2 1 7	41% 12% 6% 41%	12 3 0 11	46% 12% 0% 42%
**Number of cattle herds served by the practice**						
<100 101–200 201–300 301–400 >401	7 11 12 6 7	16% 26% 28% 14% 16%	4 4 6 1 2	24% 24% 35% 6% 11%	3 7 6 5 5	12% 27% 23% 19% 19%
**Number of farm vets in the practice**						
0-5 6–10 11–15 >15	7 23 6 7	16% 54% 14% 16%	3 9 2 3	18% 53% 11% 18%	4 14 4 4	15% 55% 15% 15%
**Species cared for**						
Farm only Mixed species	7 36	16% 84%	3 14	18% 82%	4 22	15% 85%

**Table 3:** Arwain Vet Cymru Veterinary Prescribing Champion (VPC) training schedule developed as outlined in Figure 1.

Week	Activity	Topic
1	Webinar	Welcome and introduction to antimicrobial stewardship (AMS)
2	Webinar	Encouraging behaviour change for AMS
3	Discussion Group	Developing the “Champion mindset”
4	Webinar	Prescribing rules, regulations and guidelines in farm animals
5	Webinar	Sector-specific prescribing: dairy cattle, beef cattle and sheep
6	Discussion Group	Prescribing conduct and barriers to AMS
7	Webinar	Evidence-based prescribing and practical approaches to AMS
8	Webinar	Case studies and practical examples
9	Discussion Group	The future of the VPC Network
12	Workshop	Intervention design
13	Workshop	Policy recommendations
